# The Intrinsically Disordered W Protein Is Multifunctional during Henipavirus Infection, Disrupting Host Signalling Pathways and Nuclear Import

**DOI:** 10.3390/cells9081913

**Published:** 2020-08-18

**Authors:** Sofiya Tsimbalyuk, Emily M. Cross, Mikayla Hoad, Camilla M. Donnelly, Justin A. Roby, Jade K. Forwood

**Affiliations:** School of Biomedical Sciences, Charles Sturt University, Wagga Wagga, NSW 2678, Australia; stsimbalyuk@csu.edu.au (S.T.); emcross@csu.edu.au (E.M.C.); mhoad@csu.edu.au (M.H.); cdonnelly@csu.edu.au (C.M.D.); jroby@csu.edu.au (J.A.R.)

**Keywords:** intrinsically disordered, W protein, henipaviruses, STAT, IRF-3

## Abstract

Nipah and Hendra viruses are highly pathogenic, zoonotic henipaviruses that encode proteins that inhibit the host’s innate immune response. The W protein is one of four products encoded from the P gene and binds a number of host proteins to regulate signalling pathways. The W protein is intrinsically disordered, a structural attribute that contributes to its diverse host protein interactions. Here, we review the role of W in innate immune suppression through inhibition of both pattern recognition receptor (PRR) pathways and interferon (IFN)-responsive signalling. PRR stimulation leading to activation of IRF-3 and IFN release is blocked by henipavirus W, and unphosphorylated STAT proteins are sequestered within the nucleus of host cells by W, thereby inhibiting the induction of IFN stimulated genes. We examine the critical role of nuclear transport in multiple functions of W and how specific binding of importin-alpha (Impα) isoforms, and the 14-3-3 group of regulatory proteins suggests further modulation of these processes. Overall, the disordered nature and multiple functions of W warrant further investigation to understand henipavirus pathogenesis and may reveal insights aiding the development of novel therapeutics.

## 1. Introduction

The *Paramyxoviridae* virus family contains the *Henipavirus* genus, and many other many medically significant viruses including measles virus, mumps virus, and human parainfluenza viruses, as well as Sendai virus (SeV), Newcastle disease virus (NDV), and canine distemper virus in animal species. Henipaviruses are single-stranded, negative-sense RNA viruses [1], and they include the highly pathogenic Nipah virus (NiV) and Hendra virus (HeV) [2]. Since the first HeV outbreak in the Brisbane suburb of Hendra in 1994, there have been seven positive confirmed cases in humans and 70 cases in horses, all limited to the state of Queensland and the north-east corner of the state of New South Wales, Australia [3,4]. The initial outbreak of NiV in Malaysia in 1998 and the following outbreaks in Singapore, India, Bangladesh, and the Philippines have resulted in more than 500 human cases and the mass culling of pigs in an attempt to control transmission in this reservoir species [5]. Although the community transmission of henipaviruses is low, human infection with these emerging pathogens is associated with extremely high fatality rates of 40–70% [6].

The severity of NiV and HeV infection is due in part to their ability to evade host antiviral immune responses. NiV and HeV encode multifunctional proteins that antagonise cell-intrinsic innate immune responses. The W protein (expressed from the P gene of these viruses) is intrinsically disordered and binds to multiple host targets to supress signalling pathways for interferon (IFN) production and immune responses. A refined understanding of these interactions is crucial to reveal detailed mechanisms of henipavirus pathogenesis and, in turn, may aid the development of efficacious antiviral therapeutics and attenuated vaccines. Here, we focus on the molecular interactions occurring between the W protein (a principal IFN-antagonist) and host proteins that interfere with innate immunity.

## 2. Virion Organisation

**Figure 1:**
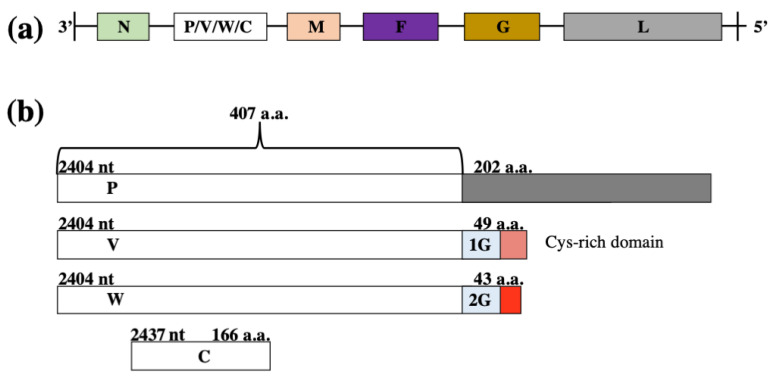

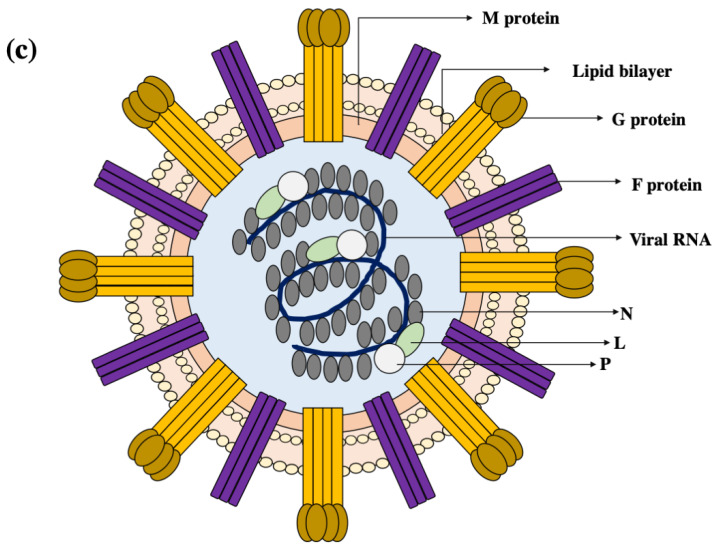
Henipavirus genome organization and virion structure (not true to scale). (**a**) The henipavirus genome is a single stranded negative polarity RNA containing six viral genes. (**b**) The P gene encodes P, V, W, and C proteins possess IFN-antagonist activity. P, V, and W share a common sequence encoding their N-terminal domain (2404–3628 nucleotides (nt)), however at site AAAAAGGGC (3619–3628 nt) RNA editing can occur with a single G-nucleotide (1G) or double G (2G) insertion shifting the reading frame resulting in unique C-terminal domains for V and W proteins respectively; C-terminus of the P protein is longer (202 aa) when compared to W and V (43 and 49 aa, respectively). (**c**) Pleomorphic virion structure is controlled by M protein which lies under the virion envelope. Attachment protein G and fusion protein F are located on the envelope surface and protrude as spikes. The viral ribonucleoprotein (RNP) core consists of a single-stranded negative sense RNA, with N, L, and P proteins required for viral transcription.

Henipaviruses are pleomorphic, non-segmented, negative-sense RNA viruses that contain a host-derived membranous envelope. The virions are variable in size and range from 40 nm to 1900 nm in diameter [7,8]. The genome of henipaviruses consists of single-stranded negative polarity RNA, and is approximately 15% longer (~18 kb in length) than other paramyxoviruses [9]. The henipavirus genome encodes six structural proteins: the attachment glycoprotein (G), matrix protein (M), phosphoprotein (P), fusion protein (F), nucleoprotein (N), and polymerase (L) (Figure 1a). The P gene encodes the P protein and three non-structural proteins; W and V through mRNA editing (resulting in unique, frame-shifted C-terminal domains) and C through an alternate open reading frame (ORF) [10] (Figure 1b).

The ribonucleoprotein (RNP) core of henipaviruses consist of a negative-sense single stranded RNA (ssRNA) molecule bound to N, L, and P proteins (Figure 1c). P and L proteins form an RNA-dependent RNA polymerase (RdRp) responsible for both transcription of viral mRNA and replication via synthesis of the positive-sense anti-genome RNA, and subsequent negative-sense genomic RNA production from this template. The L protein is responsible for the catalytic activities of the RdRp, whereas the P protein is responsible for interaction with both N-encapsidated RNA and L protein [11,12]. Genome replication of the virus occurs within the cytoplasm of the host cell, and RNP association with M and budding occurs at the cell surface to assemble infectious virions [13]. Within the virions, a shell of M protein surrounds the RNP core and makes up the inner face of the membranous envelope. As a structural protein, M contributes to both viral assembly and budding. Two glycoproteins exist on the virion outer surface; the attachment protein G assembles as homotetramer, and the fusion protein F arranges as a homotrimer [14,15,16].

## 3. The Henipavirus Replication Cycle within the Host Cell

Viral entry into the host cell is mediated by the viral G attachment protein binding to ephrin-B2 and ephrin-B3 receptors on host cells [17]. These receptors have evolved to maintain highly conserved sequences due to the necessary roles of ephrin receptors in embryogenesis and therefore display low amino acid sequence variation (3%) between dogs, horses, rats, flying foxes, and humans. This may be a factor allowing henipaviruses to infect diverse hosts [18,19]. Viral attachment to the receptor initiates a conformational change of F trimers that result in fusion of the viral envelope with the plasma membrane. The virion disassembles and releases the negative sense ssRNA genome into the cytoplasm for mRNA synthesis and protein translation.

The L protein transcribes ORF-specific mRNAs and antigenomic RNA. Viral mRNA is translated and antigenomic RNA is replicated, with production of new negative-sense genomic RNA being critically regulated by the P protein. Newly synthesised M protein translocates to the nucleus and undergoes ubiquitination [20]. It is then shuttled back to the cytoplasm where it self-assembles into an oligomeric array at the plasma membrane [20,21]. F and G proteins are translated at the endoplasmic reticulum and migrate to the plasma membrane via vesicle transport. It is thought that paramyxovirus virions assemble through an interaction of M with the cytoplasmic tails of F and G and also the RNP before budding from the host cell [22,23,24,25].

## 4. Innate Immune Antagonism and Virus Pathogenesis

The severe outcomes of HeV and NiV infections are characterized by significant respiratory distress, encephalitis, and systemic vasculitis [26,27]. Long-term neurological problems and increased risk of symptomatic relapse post-infection is an expected consequence of these viral infections. Much remains unknown about the contributions of viral factors to the respiratory and encephalitic components of henipavirus infection. The ability of henipaviruses to evade the immune system and establish systemic infection was suggested to be due to inhibition of IFN-α/β activation and signalling, with W playing a significant role [28,29]. However, in vivo NiV studies using a ferret model suggests that V plays a major role in determination of viral pathogenesis and lethality [28]. In contrast, W was established to play an important role during the inflammatory response, altering the disease course [28]. Without the ability to significantly inhibit cytokine/chemokine production, W deleted NiV mutants had reduced negative impact on the lungs and resulted in an overall extended disease course in animals. However, the prolonged period of W-deleted NiV infection resulted in increased severity of damage to the central nervous system (despite reduced lung pathology) that ultimately led to animal death by encephalitis [28,30]. Equivalent viral titres present in the lung tissue during both wild type and mutant W infection confirmed that the different respiratory tract manifestation was due to host factors rather than viral abundance [28].

## 5. W Protein Is Intrinsically Disordered and Binds Multiple Host Proteins Involved in the Suppression of Innate Immunity

**Figure 2:**
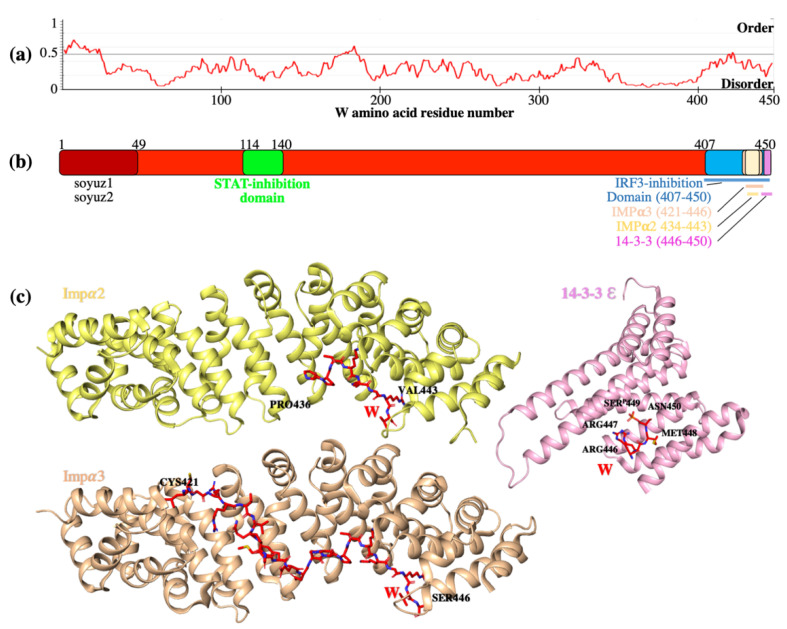
Representation of W protein domains and amino acids sequence involved in host protein interactions. (**a**) The bioinformatics program IUPred2A [40] was used to predict disordered regions across the HeV W protein sequence (UniProt accession number P0C1C6). IUPred2A uses statistical potentials to generate energy estimations of amino acid residues (whether they are likely to form favourable interactions with each other). Residues with scores <0.5 are predicted to be disordered, whereas residues scored >0.5 are likely ordered. (**b**) The N-terminal domain of W protein (grey) is an intrinsically disordered region and contains region aa 114-140 (green) that is paramount for signal transducer and activator of transcription (STAT) binding (green) and inhibition. Soyuz1 and soyuz2 regions (dark grey) are highly conserved in henipaviruses and may play a role in interferon (IFN) antagonism. The C-terminal domain of residues 408 to 450 is required for interferon regulatory factor 3 (IRF-3) inhibition (blue) and includes the Impα2 (yellow) and Impα3-binding region (orange), vital for nuclear localisation, and the 14-3-3-binding region (pink). (**c**) Solved structures of W NLS (red) in complex with binding partners: Impα1 in yellow (PDB 6BW0), Impα3 in orange (PDB 6BVV), and 14-3-3 σ in pink (PDB 6W0L).

Co-transcriptional mRNA editing of the P gene results in production of P, V, and W proteins that share the same 407 amino acids in the N-terminal domain, but each possess unique C-terminal domains [31,32]. The N-terminal domain of the henipavirus phosphoprotein is significantly longer compared to other paramyxoviruses and is intrinsically disordered, lacking defined tertiary structure [33]. P was previously found to form a tetramer through the intermolecular interactions between monomers, creating a coil-coil motif within the C-terminal domain (residues 475–578) [34,35]. However, the common N-terminal domain has only recently been partially structurally characterised as possessing several transient α-helices detected using SAXS, and no interactions in this region between any of the monomers within the tetramer were detected [35]. Similar to P, approximately 75% of the W protein sequence contains large disordered regions (LDRs) that are of negative net charge and hydrophobicity [33]. It can be suggested that the ability of the W protein to have several binding partners is due to structural flexibility of the protein in solution (Figure 2a). Curiously, sensitive sequence analysis (consisting principally of visual interrogation of multiple sequence alignments prepared from N-terminal paramyxovirus P protein homologue sequences to highlight residues conserved as motifs across several genera) revealed two conserved sequence regions named soyuz1 and soyuz2 in W among all analysed henipaviruses [36]. Soyuz1 motif is located within the first amino acids of the N-terminal domain and was proposed to likely be involved in preventing inappropriate self-assembly of RNP through interaction with the viral N protein. The conserved soyuz2 motif is present in henipavirus V, W, and P proteins and is potentially important in IFN signalling inhibition [35]. Although they are conserved across genera at the sequence level, additional research is required to evaluate the exact roles of these regions. The disordered W protein has been shown to interact with several host proteins to antagonize the innate immune response. Previously described interactions of W with human importin, STAT and 14-3-3 proteins highlight the significant contribution of W in interfering with the host antiviral state [37,38,39].

### 5.1. W Binds Impα3 and Impα4 for Nuclear Import

The discrete ability of W to localise to the nucleus is due to the presence of a strong nuclear localisation signal (NLS) within the unique C-terminal domain. Significant research has elucidated the role of this unique C-terminal domain of W in the inhibition of host innate immune responses. The importance of the C-terminal NLS in antagonist functions has been demonstrated in mutagenesis studies where the NLS mutant W behaved similar to cytoplasmic V and lost its unique ability to inhibit IFN production and downstream type I IFN signalling pathways [29,41]. The PRP19 complex (a negative regulator of p53) was identified as another W interactor, and nuclear localisation was proposed to be essential for this viral protein to functionally target PRP19 [42].

The importin family is a group of cargo-specific nuclear transport adapter proteins that contains seven isoforms of importin alpha (Impα) in humans. Through interaction with importin β, the adaptor protein in complex with its cargo protein translocate to the nucleus via the nuclear-pore complex [43,44,45]. Because the nuclear import of W protein is required for specific inhibitory functions, the basis by which W enters the nucleus is of great interest. Localization of W is predominantly nuclear due to the C-terminal NLS. Specificity of the W protein was initially described for importin α3 (Impα3) and importin α4 (Impα4) binding, however interaction with Impα1 and Impα5 was also observed in vitro to a lesser extent [29,46]. Structural studies suggested that the mechanism of specificity was due to an extended interaction interface, whereby the interactions at the major binding site interface of Impα1 and Impα3 were similar. However, an extended interface through to the minor binding site was observed for Impα3 and not Impα1 (Figure 2a) [47]. Mapping of all previous known Impα1 and Impα3 structures revealed significant structural differences close to the minor binding site of Impα3. Co-immunoprecipitation studies with point mutant and chimeric constructs demonstrated that the specificity for Impα3 was due to a specific conformation of C-terminal armadillo (ARM) repeats 7 and 8 of Impα3 [47]. The unique ARM7 and ARM8 conformation of Impα3 allowed an extended binding interface availability for NLS interactions, when compared to any other importin molecules. Importantly, other proteins that are subject to nuclear transport by Impα3 and Impα4 are outcompeted by W, leading to reduced transport and nuclear activity [48,49]. IRF-3 and other immune signalling proteins such as nuclear factor kappa B (NF-κB) show specificity for Impα3, and these findings suggest that there is potential Impα3 binding site competition between henipavirus W protein and these host antiviral transcription factors. This competitive exclusion from the nucleus may be used as a mechanism by W to suppress immune signalling. However, more work will be required to establish the exact modes of inhibition.

### 5.2. W Protein Inhibits Type I IFN Response

The antiviral innate immune response to viral infection consists of two distinct yet related signalling pathways, that have been extensively reviewed elsewhere [50,51]. Briefly, an initial pattern-recognition receptor (PRR) pathway recognises and responds to unique viral components (pathogen-associated molecular patterns; PAMPs). In *Paramyxoviridae* infections, PRRs recognising components of the single-stranded RNA (ssRNA) genome or replication intermediates, such as double-stranded RNA (dsRNA), trigger signalling cascades leading to the activation of latent transcription factors IRF-3 and NF-κB, which translocate to the nucleus, inducing the expression of inflammatory cytokines and IFN genes [52,53].

Following expression, IFNs are secreted from infected cells to induce antiviral signalling in an autocrine and paracrine manner. IFNs bind to their cognate receptors at the cell surface, leading to receptor ligation and activation of a family of cytoplasmic signalling adaptors—the Janus kinases (JAKs). JAK proteins in turn recruit and phosphorylate latent signal transducer and activator of transcription (STAT) proteins, which dimerize for translocation to the nucleus to activate IFN stimulated genes (ISGs) [54]. ISGs enact diverse antiviral roles, making IFN-responsive cells hostile to virus replication and refractory to infection [2,55].

Targeting and antagonizing the IFN pathways is thus a common strategy employed by viruses to maximise their ability to evade the immune system of the host organism and promote their replication [56]. A major mechanism by which henipaviruses successfully evade innate immune responses is by blocking virus-responsive transcriptional activation of type I IFNs (IFN-α/β). The W proteins from both HeV and NiV have been shown to interfere with PRR signalling pathways leading to inhibition of IRF-3 activation [29]. Due to the significance of IRF-3 in rapid induction of the initial antiviral response, numerous viruses encode IRF-3 inhibitors, including Ebola virus, Influenza virus, Human Papilloma Virus 16, Vaccinia virus, and Human Herpesvirus-8 (HHV-8) [57,58,59,60,61].

**Figure 3:**
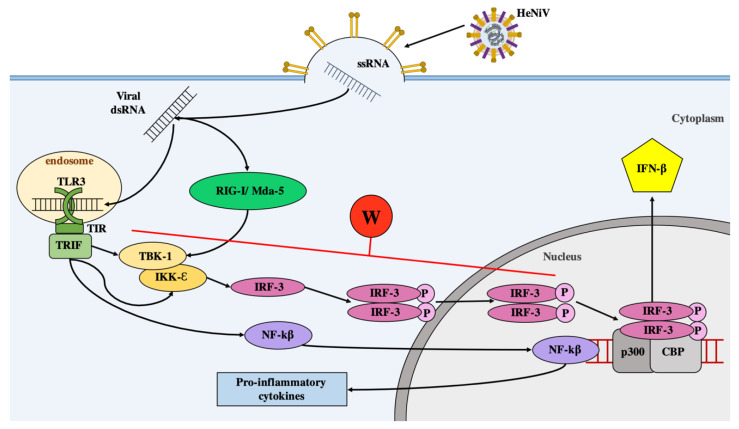
Type I IFN synthesis pathway and production of pro-inflammatory cytokines. Virus-responsive induction of the IFN-β promoter is directed by cytoplasmic helicases RIG-I and MDA5 [74]. Intracellular RNA pathogen-associated molecular patterns (PAMPs; e.g., dsRNA) are recognized by cellular pattern recognition receptors (PRRs; e.g., RIG-I, MDA5, TLR3), triggering antiviral signalling cascades. Activation of two signalling kinases TBK-1 and IKK-ε results in phosphorylation and activation of latent IRF-3 transcription factor that is subsequently translocated to the nucleus upon dimerization [67,68]. IRF-3 binds to p300 and CBP to form the DRAF1 transcription complex for IFN-β production. Cytoplasmic V protein is known to inhibit TBK-1 driven activation of IRF-3, whereas W protein successfully inhibits both TBK-1 and IKK-ε driven activation of IRF-3, with significant effects on TIR-domain-containing adapter-inducing interferon β (TRIF)-mediated activation [29].

All ssRNA viruses produce dsRNA as a product of genome replication, and this dsRNA is a potent PAMP known to trigger the innate immune system and stimulate type I IFN production within infected cells [62]. Toll-like receptor 3 (TLR3) is an evolutionarily conserved transmembrane PRR located within endosomes that is responsible for detecting endosomal viral dsRNA [63,64,65]. TLR3-dependent signalling in response to exogenous ligand (synthetic dsRNA analogue poly(I:C)) can be inhibited by NiV W protein [29]. Upon recognition of viral dsRNA, the TLR3 cytoplasmic Toll/interleukin receptor [66] domain utilises TIR-domain-containing adapter-inducing interferon β (TRIF) as the adapter for signal transduction (Figure 3) σ [66]. This interaction initiates a signalling cascade that leads to activation of TANK-binding kinase 1 (TBK-1) and inhibitor of nuclear factor kappa-B kinase subunit epsilon (IKK-ε) kinases, which in turn phosphorylate IRF-3 [67,68]. IRF-3 is a transcription factor that in its monomeric inactive form is predominantly cytoplasmic. Activation by phosphorylation results in changes to nuclear export signals and dimerization of IRF3, followed by translocation of this dimer to the nucleus [48,69]. As a result, nuclear-localized, phosphorylated IRF-3 (in conjunction with other transcription factors such as CBP and p300) binds DNA sequences within promoters to induce IFN-α/β production [70,71,72,73].

Initially, inhibition of type I IFN production by paramyxoviruses was demonstrated by the V proteins from simian virus 5 (SV5) and human parainfluenza virus 2, and by both C and V proteins from SeV [75,76,77]. Interestingly, in addition to NiV V, NiV W targets both TLR-3 and virus-mediated signalling pathways in response to SeV infection (a potent type I IFN inducer which predominantly initiates signals through RIG-I) [29].

W expression successfully lowered RIG-I-dependent SeV activation of IRF-3-responsive promoters such as ISG54 and IFN-β reporters approximately 20-fold [29]. Similar effects of inhibition were reported when intracellular transfection of a synthetic viral dsRNA analogue (poly(I:C)) was used for activation (a method known to induce MDA5-dependent antiviral signalling) [29]. W demonstrated more potent inhibitory effect on TRIF-activated expression of promoters following quantitative analysis of ISG54 and ISG56 promoter-driven mRNA when compared to V [29]. Importantly, W lacking the C-terminal domain responsible for nuclear localization performed in a similar manner to cytoplasmic V, confirming the dependence on nuclear localization for efficient W functionality [29]. Because W could impair activation of the IFN-β promoter in the presence of IKK-ε or TBK-1 (the kinases that phosphorylate and activate IRF-3), the impact of W function induced by these kinases was assessed. Results showed that W significantly inhibited activation in response to over-expression of IKK-ε or TBK-1, suggesting antagonism is mediated at or downstream of these kinases [29]. Capacity to inhibit downstream TBK-1 and IKK-ε explains and supports the observations that W impairs diverse PRR functions, including RIG-I- (SeV), MDA5- (transfected poly(I:C)), and TLR3 (exogenous poly(I:C))-dependent signalling pathways [29,37].

The influence of W on the phosphorylation of IRF-3, a transcription factor critical for induction of the IFN-β promoter downstream of TBK-1 and IKK-ε, was also assessed upon activation via overexpression of TRIF. Increasing amounts of W lead to a substantial dose-dependent reduction in phosphorylated IRF-3 within the cell, which also correlated with reduced levels of NF-κB p56 expression [29]. Effect of W on the kinase activity was sufficient to block phosphorylation and dimerization of IRF-3. The authors suggest that W may directly or indirectly affect phosphorylated IRF-3 within the nucleus, however the mechanism of inhibition is not yet clear. Finally, similar to W, IRF-3 shows preferential binding to Impα3 and α4 for nuclear localization, and a bipartite amino acid sequence corresponding to an NLS is essential for DNA-binding activity of IRF-3 [29,47,48,49] suggesting a possible mechanism for W inhibition through competitive Impα binding, blocking nuclear translocation of the active IRF-3.

Overall, the W protein blocks the dsRNA signalling pathways activated upon viral entry and replication, indicating that W targets a shared component of both activation pathways with a strong dependence on nuclear localization for some functions. The ability of W to inhibit IRF-3 activity may contribute significantly to henipavirus pathogenesis.

### 5.3. W Protein Binds Host STAT to Create High Molecular Weight Complexes that Inhibit Type I IFN Signalling 

**Figure 4:**
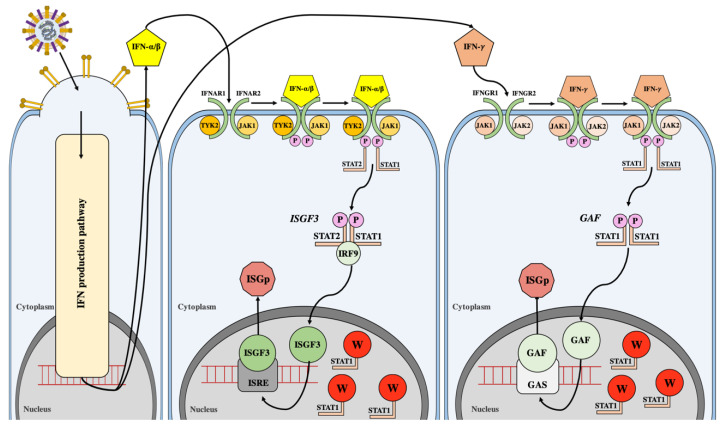
IFN signalling pathway and production of IFN-stimulated gene (ISG) products. Virus-mediated induction of the type I IFN production activates IFN signalling pathways. Type I IFNs (IFNα/β) bind cognate IFNAR receptors that activate Janus kinase (JAK) proteins JAK1 and TYK2. When activated, JAKs phosphorylate STATs. The activated STAT-1 and STAT-2 proteins form heterodimers that associate with IRF-9, creating the ISGF3 complex. ISGF3 associates with coactivators to induce the expression of genes downstream of promoters with IFN-stimulated response elements (ISREs). This upregulates many host antiviral products (ISGp). Type II interferon (IFN-γ) binds IFNGR receptors to stimulate JAK activity and phosphorylation of STAT-1 homodimers (GAF complex). GAF binds promoters of gamma-activated sequences (GAS)-associated genes that upregulate host antiviral products (ISGp). W protein binds and sequesters unphosphorylated STAT in the nucleus as a high molecular weight complex, antagonizing the function of STAT in the JAK-STAT pathway.

In response to viral infection, host antiviral defence mechanisms activate IFNs, which induce ISGs using canonical JAK-STAT pathways [54]. For type I IFNs, these cytokines bind their cognate receptor (composed of subunits IFNAR1 and IFNAR2) which induces activation of associated JAK1 and TYK2. These kinases then phosphorylate tyrosine residues on STAT-1 (Y701) and STAT-2 (Y690), allowing the formation a heterodimer through binding at C-terminal Src-homology-2 (SH2) domains. The activated STAT-1/STAT-2 heterodimer associates with IRF-9 and it is this heterotrimeric complex, designated IFN-stimulated gene factor 3 (ISGF3), which translocates to the nucleus for DNA binding. Within the nucleus, the ISGF3 complex and coactivators (CBP/p300) induce ISG expression by binding target gene IFN-stimulated response elements (ISREs) within promoters. In a similar manner, type II interferon (IFN-γ) binds to the IFNGR1 and IFNGR2 receptor subunits and activates JAK1 and JAK2. Activated JAKs phosphorylate STAT-1, which forms homodimers (gamma-activated factor or GAF) that bind gamma-activated sequences (GAS) within promoters of associated genes. ISGs consist of an array of proteins (such as PKR, OAS, and Mx proteins) that promote an active cascading host antiviral state aimed to inhibit viral replication and prevent host cell invasion (Figure 4).

As JAK-STAT signalling is critical in the host response to viral infections, many viruses have evolved mechanisms to interfere and antagonise these pathways. Rotavirus NSP1, Hepatitis C virus NS5A, and Marburg virus VP40 proteins prevent STAT phosphorylation [78,79,80]. For the *Paramyxoviridae* family, accessory proteins produced from the P gene have been implicated in altered host JAK-STAT signalling pathways and function through an assortment of molecular mechanisms. The V protein of both simian virus 5 (SV5) and mumps virus target STAT-1, whilst human parainfluenza virus type 2 (HPIV2) V protein targets STAT-2, for proteasomal degradation via V protein’s unique C-terminal domain [81,82,83].

For henipaviruses, all four P gene products are implicated with anti-IFN activity. The P protein of NiV and HeV viruses is much larger than their *Paramyxoviridae* counterparts, and although the C-terminal domain is conserved, the henipavirus proteins have an extended N-terminal domain. Interestingly for NiV V protein, STAT degradation is not observed; rather, the V protein binds STAT to form a multiprotein complex [84]. In response to cell treatment with IFN-α in the presence of NiV V protein, STAT-1 and STAT-2 show a cytoplasmic distribution within the cell. This suggested that the NiV V protein antiviral mechanism has high affinity for STAT binding that prevents tyrosine phosphorylation, nuclear accumulation, and the activation of IFN-α or IFN-β associated ISGs [84]. Unlike SV5 and HPIV2 V proteins, which display nuclear distribution, NiV V protein is almost exclusively cytoplasmic [84]. However, the V protein is efficiently shuttled from the nucleus to cytoplasm via a defined nuclear export signal interaction with CRM-1 [85]. Further to this, insights into anti-IFN activities of all NiV P/C/W/V proteins were assessed using recombinant green fluorescence protein (GFP) expressing IFN-sensitive Newcastle Disease virus (NDV) [86]. Transfection of chicken embryo fibroblast cells with NiV P/C/W/V ORF inhibited IFN production that hindered NDV-GFP replication. Overall, transfection with P/C/W/V proteins prevented the IFN antiviral state resulting in rescued ability of NDV-GFP to replicate. A shared common N-terminal domain promoted NDV-GFP replication indicating that this region may be responsible for altering IFN-α and IFN-β pathways to prevent IFN-inducible gene activation. The NiV C protein showed some anti-IFN function, however, to a lesser extent when compared to NiV V and W proteins [86]. Due to conserved ability of N-terminal domain to inhibit IFN production, it was concluded that the STAT binding domain is common for all P/W/V proteins and is present within the shared N-terminal domain of henipavirus P products.

Initially, the N-terminal STAT binding domain was found between residues 50–150, although residues 100–160 were also reported [39,85]. Subsequent to this, a stretch of amino acids between residues 114 to 140 in P/W/V proteins were shown to be required for inhibition of STAT-1 phosphorylation following IFN-β treatment [87]. Specifically, G121, G125, and G127 residues of P/V/W appear to be important for flexibility during STAT binding [87,88]. In NiV P, an aromatic amino acid at position 116 also appears to be critical for STAT-1 binding [87]. Recently, STAT-1 associated mutants of P/V/W were tested in parallel and revealed that whilst Y116E and Δ116-135 mutations of P and V proteins prevented sequestering of cytoplasmic STAT-1 and produced an altered disease course, the rNiV-P_Y116E_ and rNiV-P_Δ116-135_ viruses remained lethal in ferret models [89]. 

The W protein has been found to be the dominant STAT-1 inhibitor compared to V/P/C counterparts [39,87]. Unlike V, C, and P proteins that are mostly cytoplasmic, the W protein of NiV co-localizes to the nucleus with STAT-1 in its unphosphorylated form [39]. This indicates that W either binds STAT-1 in the nucleus, preventing its nuclear export and recycling in the JAK-STAT pathway, or transports latent (and unphosphorylated) STAT-1 directly to the nucleus where W then sequesters it as a high molecular weight non-functional complex (Figure 4) [39]. The W protein has been shown to play the more prominent role in evading host innate immunity by supressing the IFN induced JAK-STAT pathway and may ultimately affect disease course.

### 5.4. W Binds Host 14-3-3 Proteins to Modulate Diverse Host Signalling Pathways

Interactome studies investigating HeV and NiV W and host proteins have identified interactions with several members of the 14-3-3 family [90]. The 14-3-3 family is comprised of small, highly conserved proteins that are crucial for the regulation of intracellular signalling pathways by altering activity, intracellular localization and post-translational modifications of proteins involved [91]. There are seven known isoforms of mammalian 14-3-3 proteins: Beta (β), Epsilon (ε), Gamma (γ), Eta (η), Sigma (σ), Tau (τ) and Zeta (ζ) [92]. All 14-3-3 proteins share a similar structure with a conserved N-terminal dimerization domain and a region for target protein binding [91,93]. 14-3-3 proteins are able to associate with a wide range of different molecules, primarily due to specific phosphoserine/phosphothreonine binding activity by high affinity phosphorylation-dependent binding motifs harboured on the binding groove of 14-3-3 isotypes [94,95]. Moreover, 14-3-3 interactions in innate immune signaling have been extensively described [29,56,57]. For detailed discussion of the many roles of the 14-3-3 family in antiviral signalling, we direct the reader to several excellent recent reviews [94,96,97]. Briefly, 14-3-3 involved in the translocation of RIG-I to organelles that mediate downstream RIG-I signalling, resulting in interferon production. For example, 14-3-3ϵ and 14-3-3η promote the translocation of RIG-I and MDA5 to mitochondria respectively [98].

Using co-immunoprecipitation (co-IP) assays, all 14-3-3 isoforms were shown to interact with W protein, although to different extents [37]. Through examination of different domain constructs and mutation studies where key suspected residues were substituted with alanine, it was found that the W interaction with 14-3-3 was mediated through a phosphorylated Ser449 residue in the unique C-terminus of W [37]. This serine is the penultimate amino acid residue in both the NiV and HeV W sequence and represents the basis for a mode III type binding. Mode III binding is initiated by interaction of a 14-3-3 protein with the C-terminal domain of a protein containing a phosphorylated serine. Supporting this binding data, a crystal structure of the NiV W (spanning residues 446-RRMpSN-450) confirmed a mode III binding motif (Figure 2a) [37]. NiV W protein has been shown to be bound in extended unfolded form. The binding sequences of 14-3-3 and Impα on W proteins are in close proximity, suggesting the possibility of competition between binding partners. However, competition co-IPs demonstrated that 14-3-3 did not disrupt Impα interaction with NiV W, suggesting that 14-3-3 is unlikely to be a regulator of W nuclear import. Conversely, Impα was shown to outcompete 14-3-3 for interaction with NiV W, suggesting a possible regulatory role of Impα for NiV W:14-3-3 interaction. 14-3-3ε facilitates host immune RIG-I signalling through direct interaction and activation of MDA5 resulting in enhancement of the IFNβ promoter activities [97]. Due to the the essential role of 14-3-3 isoforms in RIG-I/MDA5 signalling and W ability to inhibit innate immunity, the possibility of W having antagonist function against this pathway via 14-3-3 proteins was assessed. IFNβ promoter luciferase assays using SeV infection have shown the inhibition if RIG-I signalling by NiV W protein irrespective of 14-3-3 interaction. Since 14-3-3 ε and RIG-I interaction is cytoplasmic, expression of W was shifted to cytoplasm, by mutating NLS sequence, to further investigate W effect on 14-3-3 function. However, no difference in activity was observed when compared to WT NiV W [37]. Results indicated that the 14-3-3:NiV W interaction is not required for inhibition of RIG-I/MDA5 signalling pathway of IFN production by the W protein. However, the NiV W interaction with 14-3-3 was shown to modulate diverse host signalling pathways including innate immune related host gene expression, in the context of RNA virus infection. [37]. The transcriptional profile of cells overexpressing NiV W protein (or a mutant W protein lacking 14-3-3 interaction) was assessed via next generation sequencing. Analysis of the differentially expressed genes (DEG) revealed that the downregulated DEG subset was enriched for genes that are controlled by immune-related transcription factors such as STAT-1 and IRF-1. These data strongly suggest that NiV W interaction with 14-3-3 inhibits the activation and/or signalling of immune-related transcription factors, leading to a footprint of downregulated immune genes [37].

## 6. Conclusions

Significant work has been conducted to better understand W IFN-antagonist activity in HeV and NiV infection, with a major focus on IRF-3 inhibition, STAT sequestration, and nuclear translocation via importin alpha proteins. Recent work describing 14-3-3 and W interaction has suggested potential new functions of W that may contribute to its role in henipavirus pathogenesis. Additional research is required to further understand and establish the exact interfaces of W and host proteins that contribute to regulation of host innate immune responses. W–host interactions described in this review may present targets for the development of new antivirals.

## References

[1] Rima B., Balkema-Buschmann A., Dundon W.G., Duprex P., Easton A., Fouchier R., Kurath G., Lamb R., Lee B., Rota P. (2019). ICTV Virus Taxonomy Profile: Paramyxoviridae. J. Gen. Virol..

[2] Schneider W.M., Chevillotte M.D., Rice C.M. (2014). Interferon-stimulated genes: A complex web of host defenses. Annu. Rev. Immunol..

[3] Murray K., Selleck P., Hooper P., Hyatt A., Gould A., Gleeson L., Westbury H., Hiley L., Selvey L., Rodwell B. (1995). A morbillivirus that caused fatal disease in horses and humans. Science.

[4] Field H.E. (2016). Hendra virus ecology and transmission. Curr. Opin. Virol..

[5] Ang B.S.P., Lim T.C.C., Wang L. (2018). Nipah Virus Infection. J. Clin. Microbiol..

[6] Luby S.P., Hossain M.J., Gurley E.S., Ahmed B.N., Banu S., Khan S.U., Homaira N., Rota P.A., Rollin P.E., Comer J.A. (2009). Recurrent zoonotic transmission of Nipah virus into humans, Bangladesh, 2001–2007. Emerg. Infect. Dis..

[7] Goldsmith C.S., Whistler T., Rollin P.E., Ksiazek T.G., Rota P.A., Bellini W.J., Daszak P., Wong K.T., Shieh W.J., Zaki S.R. (2003). Elucidation of Nipah virus morphogenesis and replication using ultrastructural and molecular approaches. Virus Res..

[8] Patch J.R., Crameri G., Wang L.-F., Eaton B.T., Broder C.C. (2007). Quantitative analysis of Nipah virus proteins released as virus-like particles reveals central role for the matrix protein. Virol. J..

[9] Wang L.F., Yu M., Hansson E., Pritchard L.I., Shiell B., Michalski W.P., Eaton B.T. (2000). The exceptionally large genome of Hendra virus: Support for creation of a new genus within the family Paramyxoviridae. J. Virol..

[10] Enchéry F., Horvat B. (2017). Understanding the Interaction between Henipaviruses and Their Natural Host, Fruit Bats: Paving the Way Toward Control of Highly Lethal Infection in Humans. Int. Rev. Immunol..

[11] Hausmann S., Garcin D., Delenda C., Kolakofsky D. (1999). The versatility of paramyxovirus RNA polymerase stuttering. J. Virol..

[12] Lo M.K., Harcourt B.H., Mungall B.A., Tamin A., Peeples M.E., Bellini W.J., Rota P.A. (2009). Determination of the henipavirus phosphoprotein gene mRNA editing frequencies and detection of the C, V and W proteins of Nipah virus in virus-infected cells. J. Gen. Virol..

[13] Battisti A.J., Meng G., Winkler D.C., McGinnes L.W., Plevka P., Steven A.C., Morrison T.G., Rossmann M.G. (2012). Structure and assembly of a paramyxovirus matrix protein. Proc. Natl. Acad. Sci. USA.

[14] Singh M., Berger B., Kim P.S. (1999). LearnCoil-VMF: Computational evidence for coiled-coil-like motifs in many viral membrane-fusion proteins. J. Mol. Biol..

[15] Langedijk J.P., Daus F.J., van Oirschot J.T. (1997). Sequence and structure alignment of Paramyxoviridae attachment proteins and discovery of enzymatic activity for a morbillivirus hemagglutinin. J. Virol..

[16] Crennell S., Takimoto T., Portner A., Taylor G. (2000). Crystal structure of the multifunctional paramyxovirus hemagglutinin-neuraminidase. Nat. Struct. Biol..

[17] Negrete O.A., Levroney E.L., Aguilar H.C., Bertolotti-Ciarlet A., Nazarian R., Tajyar S., Lee B. (2005). EphrinB2 is the entry receptor for Nipah virus, an emergent deadly paramyxovirus. Nature.

[18] Aguilar H.C., Iorio R.M. (2012). Henipavirus membrane fusion and viral entry. Curr. Top. Microbiol. Immunol..

[19] Pernet O., Wang Y.E., Lee B. (2012). Henipavirus receptor usage and tropism. Curr. Top. Microbiol. Immunol..

[20] Pentecost M., Vashisht A.A., Lester T., Voros T., Beaty S.M., Park A., Wang Y.E., Yun T.E., Freiberg A.N., Wohlschlegel J.A. (2015). Evidence for Ubiquitin-Regulated Nuclear and Subnuclear Trafficking among Paramyxovirinae Matrix Proteins. PLoS Pathog..

[21] Wang Y.E., Park A., Lake M., Pentecost M., Torres B., Yun T.E., Wolf M.C., Holbrook M.R., Freiberg A.N., Lee B. (2010). Ubiquitin-regulated nuclear-cytoplasmic trafficking of the Nipah virus matrix protein is important for viral budding. PLoS Pathog..

[22] Waning D.L., Schmitt A.P., Leser G.P., Lamb R.A. (2002). Roles for the cytoplasmic tails of the fusion and hemagglutinin-neuraminidase proteins in budding of the paramyxovirus simian virus 5. J. Virol..

[23] Pantua H.D., McGinnes L.W., Peeples M.E., Morrison T.G. (2006). Requirements for the assembly and release of Newcastle disease virus-like particles. J. Virol..

[24] Harrison M.S., Sakaguchi T., Schmitt A.P. (2010). Paramyxovirus assembly and budding: Building particles that transmit infections. Int. J. Biochem. Cell Biol..

[25] Ghildyal R., Mills J., Murray M., Vardaxis N., Meanger J. (2002). Respiratory syncytial virus matrix protein associates with nucleocapsids in infected cells. J. Gen. Virol..

[26] Goh K.J., Tan C.T., Chew N.K., Tan P.S., Kamarulzaman A., Sarji S.A., Wong K.T., Abdullah B.J., Chua K.B., Lam S.K. (2000). Clinical features of Nipah virus encephalitis among pig farmers in Malaysia. N. Engl. J. Med..

[27] Wong K.T., Shieh W.J., Kumar S., Norain K., Abdullah W., Guarner J., Goldsmith C.S., Chua K.B., Lam S.K., Tan C.T. (2002). Nipah virus infection: Pathology and pathogenesis of an emerging paramyxoviral zoonosis. Am. J. Pathol..

[28] Satterfield B.A., Cross R.W., Fenton K.A., Agans K.N., Basler C.F., Geisbert T.W., Mire C.E. (2015). The immunomodulating V and W proteins of Nipah virus determine disease course. Nat. Commun..

[29] Shaw M.L., Cardenas W.B., Zamarin D., Palese P., Basler C.F. (2005). Nuclear localization of the Nipah virus W protein allows for inhibition of both virus- and toll-like receptor 3-triggered signaling pathways. J. Virol..

[30] Satterfield B.A., Cross R.W., Fenton K.A., Borisevich V., Agans K.N., Deer D.J., Graber J., Basler C.F., Geisbert T.W., Mire C.E. (2016). Nipah Virus C and W Proteins Contribute to Respiratory Disease in Ferrets. J. Virol..

[31] Kolakofsky D., Roux L., Garcin D., Ruigrok R.W.H. (2005). Paramyxovirus mRNA editing, the “rule of six” and error catastrophe: A hypothesis. J. Gen. Virol..

[32] Curran J., Kolakofsky D. (1990). Sendai virus P gene produces multiple proteins from overlapping open reading frames. Enzyme.

[33] Karlin D., Longhi S., Receveur V., Canard B. (2002). The N-terminal domain of the phosphoprotein of Morbilliviruses belongs to the natively unfolded class of proteins. Virology.

[34] Curran J., Boeck R., Lin-Marq N., Lupas A., Kolakofsky D. (1995). Paramyxovirus phosphoproteins form homotrimers as determined by an epitope dilution assay, via predicted coiled coils. Virology.

[35] Jensen M.R., Yabukarski F., Communie G., Condamine E., Mas C., Volchkova V., Tarbouriech N., Bourhis J.M., Volchkov V., Blackledge M. (2020). Structural Description of the Nipah Virus Phosphoprotein and Its Interaction with STAT1. Biophys. J..

[36] Karlin D., Belshaw R. (2012). Detecting remote sequence homology in disordered proteins: Discovery of conserved motifs in the N-termini of Mononegavirales phosphoproteins. PLoS ONE.

[37] Edwards M.R., Hoad M., Tsimbalyuk S., Menicucci A.R., Messaoudi I., Forwood J.K., Basler C.F. (2020). Henipavirus W proteins interact with 14-3-3 to modulate host gene expression. J. Virol..

[38] Parisien J.P., Lau J.F., Rodriguez J.J., Ulane C.M., Horvath C.M. (2002). Selective STAT protein degradation induced by paramyxoviruses requires both STAT1 and STAT2 but is independent of alpha/beta interferon signal transduction. J. Virol..

[39] Shaw M.L., García-Sastre A., Palese P., Basler C.F. (2004). Nipah virus V and W proteins have a common STAT1-binding domain yet inhibit STAT1 activation from the cytoplasmic and nuclear compartments, respectively. J. Virol..

[40] Erdős G., Dosztányi Z. (2020). Analyzing Protein Disorder with IUPred2A. Curr. Protoc. Bioinform..

[41] Lo M.K., Miller D., Aljofan M., Mungall B.A., Rollin P.E., Bellini W.J., Rota P.A. (2010). Characterization of the antiviral and inflammatory responses against Nipah virus in endothelial cells and neurons. Virology.

[42] Martinez-Gil L., Vera-Velasco N.M., Mingarro I. (2017). Exploring the Human-Nipah Virus Protein-Protein Interactome. J. Virol..

[43] Lange A., Mills R.E., Lange C.J., Stewart M., Devine S.E., Corbett A.H. (2007). Classical nuclear localization signals: Definition, function, and interaction with importin alpha. J. Biol. Chem..

[44] Stewart M. (2007). Molecular mechanism of the nuclear protein import cycle. Nat. Rev. Mol. Cell Biol..

[45] Goldfarb D.S., Corbett A.H., Mason D.A., Harreman M.T., Adam S.A. (2004). Importin alpha: A multipurpose nuclear-transport receptor. Trends Cell Biol..

[46] Garcia-Sastre A. (2002). Mechanisms of inhibition of the host interferon alpha/beta-mediated antiviral responses by viruses. Microbes Infect..

[47] Smith K.M., Tsimbalyuk S., Edwards M.R., Cross E.M., Batra J., Soares da Costa T.P., Aragao D., Basler C.F., Forwood J.K. (2018). Structural basis for importin alpha 3 specificity of W proteins in Hendra and Nipah viruses. Nat. Commun..

[48] Kumar K.P., McBride K.M., Weaver B.K., Dingwall C., Reich N.C. (2000). Regulated nuclear-cytoplasmic localization of interferon regulatory factor 3, a subunit of double-stranded RNA-activated factor 1. Mol. Cell Biol..

[49] Zhu M., Fang T., Li S., Meng K., Guo D. (2015). Bipartite Nuclear Localization Signal Controls Nuclear Import and DNA-Binding Activity of IFN Regulatory Factor 3. J. Immunol..

[50] Seth R.B., Sun L., Chen Z.J. (2006). Antiviral innate immunity pathways. Cell Res..

[51] Braciale T.J., Hahn Y.S. (2013). Immunity to viruses. Immunol. Rev..

[52] Ivashkiv L.B., Donlin L.T. (2014). Regulation of type I interferon responses. Nat. Rev. Immunol..

[53] Gay N.J., Symmons M.F., Gangloff M., Bryant C.E. (2014). Assembly and localization of Toll-like receptor signalling complexes. Nat. Rev. Immunol..

[54] Chiang H.S., Liu H.M. (2018). The Molecular Basis of Viral Inhibition of IRF- and STAT-Dependent Immune Responses. Front. Immunol..

[55] Liao Y., Goraya M.U., Yuan X., Zhang B., Chiu S.H., Chen J.L. (2019). Functional Involvement of Interferon-Inducible Transmembrane Proteins in Antiviral Immunity. Front. Microbiol..

[56] Basler C.F., Garcia-Sastre A. (2002). Viruses and the type I interferon antiviral system: Induction and evasion. Int. Rev. Immunol..

[57] Basler C.F., Mikulasova A., Martinez-Sobrido L., Paragas J., Mühlberger E., Bray M., Klenk H.D., Palese P., García-Sastre A. (2003). The Ebola virus VP35 protein inhibits activation of interferon regulatory factor 3. J. Virol..

[58] Ronco L.V., Karpova A.Y., Vidal M., Howley P.M. (1998). Human papillomavirus 16 E6 oncoprotein binds to interferon regulatory factor-3 and inhibits its transcriptional activity. Genes Dev..

[59] Lin R., Genin P., Mamane Y., Sgarbanti M., Battistini A., Harrington W.J., Barber G.N., Hiscott J. (2001). HHV-8 encoded vIRF-1 represses the interferon antiviral response by blocking IRF-3 recruitment of the CBP/p300 coactivators. Oncogene.

[60] Talon J., Horvath C.M., Polley R., Basler C.F., Muster T., Palese P., García-Sastre A. (2000). Activation of interferon regulatory factor 3 is inhibited by the influenza A virus NS1 protein. J. Virol..

[61] Xiang Y., Condit R.C., Vijaysri S., Jacobs B., Williams B.R., Silverman R.H. (2002). Blockade of interferon induction and action by the E3L double-stranded RNA binding proteins of vaccinia virus. J. Virol..

[62] Jacobs B.L., Langland J.O. (1996). When two strands are better than one: The mediators and modulators of the cellular responses to double-stranded RNA. Virology.

[63] Akira S., Takeda K. (2004). Toll-like receptor signalling. Nat. Rev. Immunol..

[64] Hoffmann J.A. (2003). The immune response of Drosophila. Nature.

[65] Alexopoulou L., Holt A.C., Medzhitov R., Flavell R.A. (2001). Recognition of double-stranded RNA and activation of NF-kappaB by Toll-like receptor 3. Nature.

[66] Maes T., Mascaró C., Tirapu I., Estiarte A., Ciceri F., Lunardi S., Guibourt N., Perdones A., Lufino M.M.P., Somervaille T.C.P. (2018). ORY-1001, a Potent and Selective Covalent KDM1A Inhibitor, for the Treatment of Acute Leukemia. Cancer Cell.

[67] Fitzgerald K.A., McWhirter S.M., Faia K.L., Rowe D.C., Latz E., Golenbock D.T., Coyle A.J., Liao S.M., Maniatis T. (2003). IKKepsilon and TBK1 are essential components of the IRF3 signaling pathway. Nat. Immunol..

[68] Sharma S., tenOever B.R., Grandvaux N., Zhou G.P., Lin R., Hiscott J. (2003). Triggering the interferon antiviral response through an IKK-related pathway. Science.

[69] Lin R., Heylbroeck C., Pitha P.M., Hiscott J. (1998). Virus-dependent phosphorylation of the IRF-3 transcription factor regulates nuclear translocation, transactivation potential, and proteasome-mediated degradation. Mol. Cell Biol..

[70] Yoneyama M., Suhara W., Fukuhara Y., Fukuda M., Nishida E., Fujita T. (1998). Direct triggering of the type I interferon system by virus infection: Activation of a transcription factor complex containing IRF-3 and CBP/p300. EMBO J..

[71] Grandvaux N., Servant M.J., tenOever B., Sen G.C., Balachandran S., Barber G.N., Lin R., Hiscott J. (2002). Transcriptional profiling of interferon regulatory factor 3 target genes: Direct involvement in the regulation of interferon-stimulated genes. J. Virol..

[72] Peters K.L., Smith H.L., Stark G.R., Sen G.C. (2002). IRF-3-dependent, NFkappa B- and JNK-independent activation of the 561 and IFN-beta genes in response to double-stranded RNA. Proc. Natl. Acad. Sci. USA.

[73] Sato M., Suemori H., Hata N., Asagiri M., Ogasawara K., Nakao K., Nakaya T., Katsuki M., Noguchi S., Tanaka N. (2000). Distinct and essential roles of transcription factors IRF-3 and IRF-7 in response to viruses for IFN-alpha/beta gene induction. Immunity.

[74] Iampietro M., Aurine N., Dhondt K.P., Dumont C., Pelissier R., Spanier J., Vallve A., Raoul H., Kalinke U., Horvat B. (2020). Control of Nipah Virus Infection in Mice by the Host Adaptors Mitochondrial Antiviral Signaling Protein (MAVS) and Myeloid Differentiation Primary Response 88 (MyD88). J. Infect. Dis..

[75] He B., Paterson R.G., Stock N., Durbin J.E., Durbin R.K., Goodbourn S., Randall R.E., Lamb R.A. (2002). Recovery of paramyxovirus simian virus 5 with a V protein lacking the conserved cysteine-rich domain: The multifunctional V protein blocks both interferon-beta induction and interferon signaling. Virology.

[76] Poole E., He B., Lamb R.A., Randall R.E., Goodbourn S. (2002). The V proteins of simian virus 5 and other paramyxoviruses inhibit induction of interferon-beta. Virology.

[77] Komatsu T., Takeuchi K., Yokoo J., Gotoh B. (2004). C and V proteins of Sendai virus target signaling pathways leading to IRF-3 activation for the negative regulation of interferon-beta production. Virology.

[78] Kumthip K., Chusri P., Jilg N., Zhao L., Fusco D.N., Zhao H., Goto K., Cheng D., Schaefer E.A., Zhang L. (2012). Hepatitis C virus NS5A disrupts STAT1 phosphorylation and suppresses type I interferon signaling. J. Virol..

[79] Sen A., Rott L., Phan N., Mukherjee G., Greenberg H.B. (2014). Rotavirus NSP1 Protein Inhibits Interferon-Mediated STAT1 Activation. J. Virol..

[80] Valmas C., Grosch M.N., Schümann M., Olejnik J., Martinez O., Best S.M., Krähling V., Basler C.F., Mühlberger E. (2010). Marburg virus evades interferon responses by a mechanism distinct from ebola virus. PLoS Pathog..

[81] Didcock L., Young D.F., Goodbourn S., Randall R.E. (1999). The V protein of simian virus 5 inhibits interferon signalling by targeting STAT1 for proteasome-mediated degradation. J. Virol..

[82] Parisien J.-P., Lau J.F., Rodriguez J.J., Sullivan B.M., Moscona A., Parks G.D., Lamb R.A., Horvath C.M. (2001). The V Protein of Human Parainfluenza Virus 2 Antagonizes Type I Interferon Responses by Destabilizing Signal Transducer and Activator of Transcription 2. Virology.

[83] Kubota T., Yokosawa N., Yokota S.-I., Fujii N. (2001). C Terminal CYS-RICH Region of Mumps Virus Structural V Protein Correlates with Block of Interferon α and γ Signal Transduction Pathway through Decrease of STAT 1-α. Biochem. Biophys. Res. Commun..

[84] Rodriguez J.J., Parisien J.-P., Horvath C.M. (2002). Nipah virus V protein evades alpha and gamma interferons by preventing STAT1 and STAT2 activation and nuclear accumulation. J. Virol..

[85] Rodriguez J.J., Cruz C.D., Horvath C.M. (2004). Identification of the nuclear export signal and STAT-binding domains of the Nipah virus V protein reveals mechanisms underlying interferon evasion. J. Virol..

[86] Park M.-S., Shaw M.L., Muñoz-Jordan J., Cros J.F., Nakaya T., Bouvier N., Palese P., García-Sastre A., Basler C.F. (2003). Newcastle disease virus (NDV)-based assay demonstrates interferon-antagonist activity for the NDV V protein and the Nipah virus V, W, and C proteins. J. Virol..

[87] Ciancanelli M.J., Volchkova V.A., Shaw M.L., Volchkov V.E., Basler C.F. (2009). Nipah virus sequesters inactive STAT1 in the nucleus via a P gene-encoded mechanism. J. Virol..

[88] Hagmaier K., Stock N., Goodbourn S., Wang L.-F., Randall R. (2006). A single amino acid substitution in the V protein of Nipah virus alters its ability to block interferon signalling in cells from different species. J. Gen. Virol..

[89] Satterfield B.A., Borisevich V., Foster S.L., Rodriguez S.E., Cross R.W., Fenton K.A., Agans K.N., Basler C.F., Geisbert T.W., Mire C.E. (2019). Antagonism of STAT1 by Nipah virus P gene products modulates disease course but not lethal outcome in the ferret model. Sci. Rep..

[90] Pichlmair A., Kandasamy K., Alvisi G., Mulhern O., Sacco R., Habjan M., Binder M., Stefanovic A., Eberle C.A., Goncalves A. (2012). Viral immune modulators perturb the human molecular network by common and unique strategies. Nature.

[91] Aghazadeh Y., Papadopoulos V. (2016). The role of the 14-3-3 protein family in health, disease, and drug development. Drug Discov. Today.

[92] Fu H., Subramanian R.R., Masters S.C. (2000). 14-3-3 proteins: Structure, function, and regulation. Annu. Rev. Pharmacol. Toxicol..

[93] Tzivion G., Shen Y.H., Zhu J. (2001). 14-3-3 proteins; bringing new definitions to scaffolding. Oncogene.

[94] Dougherty M.K., Morrison D.K. (2004). Unlocking the code of 14-3-3. J. Cell Sci..

[95] Wang B., Yang H., Liu Y.C., Jelinek T., Zhang L., Ruoslahti E., Fu H. (1999). Isolation of high-affinity peptide antagonists of 14-3-3 proteins by phage display. Biochemistry.

[96] Lin J.P., Fan Y.K., Liu H.M. (2019). The 14-3-3η chaperone protein promotes antiviral innate immunity via facilitating MDA5 oligomerization and intracellular redistribution. PLoS Pathog..

[97] Liu H.M., Loo Y.M., Horner S.M., Zornetzer G.A., Katze M.G., Gale M. (2012). The mitochondrial targeting chaperone 14-3-3ε regulates a RIG-I translocon that mediates membrane association and innate antiviral immunity. Cell Host Microbe.

[98] Riedl W., Acharya D., Lee J.H., Liu G., Serman T., Chiang C., Chan Y.K., Diamond M.S., Gack M.U. (2019). Zika Virus NS3 Mimics a Cellular 14-3-3-Binding Motif to Antagonize RIG-I- and MDA5-Mediated Innate Immunity. Cell Host Microbe.

