# Correction: Tsimbalyuk et al. The Intrinsically Disordered W Protein Is Multifunctional during Henipavirus Infection, Disrupting Host Signalling Pathways and Nuclear Import. *Cells* **2020**, *9*, 1913

**DOI:** 10.3390/cells11081290

**Published:** 2022-04-11

**Authors:** Sofiya Tsimbalyuk, Emily M. Cross, Mikayla Hoad, Camilla M. Donnelly, Justin A. Roby, Jade K. Forwood

**Affiliations:** School of Biomedical Sciences, Charles Sturt University, Wagga Wagga, NSW 2678, Australia; stsimbalyuk@csu.edu.au (S.T.); emcross@csu.edu.au (E.M.C.); mhoad@csu.edu.au (M.H.); cdonnelly@csu.edu.au (C.M.D.); jroby@csu.edu.au (J.A.R.)

## Text Correction

The authors would like to make the following corrections to the published paper [1]: 

There are some mistakes in the order of references and their citations in the original article due to bibliography software errors [1]. In the original publication, Shaw, M.L. et al., 2004 was reference 20 in the reference list, and now that we have updated the reference list as below (Reference List Correction), it has become reference 39. Consequently, we have also updated the citation number in *Section 5.3. W Protein Binds Host STAT to Create High Molecular Weight Complexes that Inhibit Type I IFN Signalling*, *page 10,* which should now read:

The W protein has been found to be the dominant STAT-1 inhibitor compared to V/P/C counterparts [39,87]. Unlike V, C, and P proteins that are mostly cytoplasmic, the W protein of NiV co-localizes to the nucleus with STAT-1 in its unphosphorylated form [39]. This indicates that W either binds STAT-1 in the nucleus, preventing its nuclear export and recycling in the JAK-STAT pathway, or transports latent (and unphosphorylated) STAT-1 directly to the nucleus where W then sequesters it as a high molecular weight non-functional complex (Figure 4) [39].

## Reference List Correction

There was an error in the original publication. Previous references 20–34 should move to be references 39–53 in the correct version. Previous references 35–53 should move to be references 20–38 in the correct version.

A correction has been made to *References*, *pages 12–14.*

20. Pentecost, M.; Vashisht, A.A.; Lester, T.; Voros, T.; Beaty, S.M.; Park, A.; Wang, Y.E.; Yun, T.E.; Freiberg, A.N.; Wohlschlegel, J.A.; et al. Evidence for Ubiquitin-Regulated Nuclear and Subnuclear Trafficking among Paramyxovirinae Matrix Proteins. *PLoS Pathog.* **2015**, *11*, e1004739.
21. Wang, Y.E.; Park, A.; Lake, M.; Pentecost, M.; Torres, B.; Yun, T.E.; Wolf, M.C.; Holbrook, M.R.; Freiberg, A.N.; Lee, B. Ubiquitin-regulated nuclear-cytoplasmic trafficking of the Nipah virus matrix protein is important for viral budding. *PLoS Pathog.* **2010**, *6*, e1001186.
22. Waning, D.L.; Schmitt, A.P.; Leser, G.P.; Lamb, R.A. Roles for the cytoplasmic tails of the fusion and hemagglutinin-neuraminidase proteins in budding of the paramyxovirus simian virus 5. *J. Virol.* **2002**, *76*, 9284–9297.
23. Pantua, H.D.; McGinnes, L.W.; Peeples, M.E.; Morrison, T.G. Requirements for the assembly and release of Newcastle disease virus-like particles. *J. Virol.* **2006**, *80*, 11062–11073.
24. Harrison, M.S.; Sakaguchi, T.; Schmitt, A.P. Paramyxovirus assembly and budding: Building particles that transmit infections. *Int. J. Biochem. Cell Biol.* **2010**, *42*, 1416–1429.
25. Ghildyal, R.; Mills, J.; Murray, M.; Vardaxis, N.; Meanger, J. Respiratory syncytial virus matrix protein associates with nucleocapsids in infected cells. *J. Gen. Virol.* **2002**, *83*, 753–757.
26. Goh, K.J.; Tan, C.T.; Chew, N.K.; Tan, P.S.; Kamarulzaman, A.; Sarji, S.A.; Wong, K.T.; Abdullah, B.J.; Chua, K.B.; Lam, S.K. Clinical features of Nipah virus encephalitis among pig farmers in Malaysia. *N. Engl. J. Med.* **2000**, *342*, 1229–1235.
27. Wong, K.T.; Shieh, W.J.; Kumar, S.; Norain, K.; Abdullah, W.; Guarner, J.; Goldsmith, C.S.; Chua, K.B.; Lam, S.K.; Tan, C.T.; et al. Nipah virus infection: Pathology and pathogenesis of an emerging paramyxoviral zoonosis. *Am. J. Pathol.* **2002**, *161*, 2153–2167.
28. Satterfield, B.A.; Cross, R.W.; Fenton, K.A.; Agans, K.N.; Basler, C.F.; Geisbert, T.W.; Mire, C.E. The immunomodulating V and W proteins of Nipah virus determine disease course. *Nat. Commun.* **2015**, *6*, 7483.
29. Shaw, M.L.; Cardenas, W.B.; Zamarin, D.; Palese, P.; Basler, C.F. Nuclear localization of the Nipah virus W protein allows for inhibition of both virus- and toll-like receptor 3-triggered signaling pathways. *J. Virol.* **2005**, *79*, 6078–6088.
30. Satterfield, B.A.; Cross, R.W.; Fenton, K.A.; Borisevich, V.; Agans, K.N.; Deer, D.J.; Graber, J.; Basler, C.F.; Geisbert, T.W.; Mire, C.E. Nipah Virus C and W Proteins Contribute to Respiratory Disease in Ferrets. *J. Virol.* **2016**, *90*, 6326–6343.
31. Kolakofsky, D.; Roux, L.; Garcin, D.; Ruigrok, R.W.H. Paramyxovirus mRNA editing, the “rule of six” and error catastrophe: A hypothesis. *J. Gen. Virol.* **2005**, *86*, 1869–1877.
32. Curran, J.; Kolakofsky, D. Sendai virus P gene produces multiple proteins from overlapping open reading frames. *Enzyme* **1990**, *44*, 244–249.
33. Karlin, D.; Longhi, S.; Receveur, V.; Canard, B. The N-terminal domain of the phosphoprotein of Morbilliviruses belongs to the natively unfolded class of proteins. *Virology* **2002**, *296*, 251–262.
34. Curran, J.; Boeck, R.; Lin-Marq, N.; Lupas, A.; Kolakofsky, D. Paramyxovirus phosphoproteins form homotrimers as determined by an epitope dilution assay, via predicted coiled coils. *Virology* **1995**, *214*, 139–149.
35. Jensen, M.R.; Yabukarski, F.; Communie, G.; Condamine, E.; Mas, C.; Volchkova, V.; Tarbouriech, N.; Bourhis, J.M.; Volchkov, V.; Blackledge, M.; et al. Structural Description of the Nipah Virus Phosphoprotein and Its Interaction with STAT1. *Biophys. J.* **2020**. https://doi.org/10.1016/j.bpj.2020.04.010.
36. Karlin, D.; Belshaw, R. Detecting remote sequence homology in disordered proteins: Discovery of conserved motifs in the N-termini of Mononegavirales phosphoproteins. *PLoS ONE* **2012**, *7*, e31719.
37. Edwards, M.R.; Hoad, M.; Tsimbalyuk, S.; Menicucci, A.R.; Messaoudi, I.; Forwood, J.K.; Basler, C.F. Henipavirus W proteins interact with 14-3-3 to modulate host gene expression. *J. Virol.* **2020**. https://doi.org/10.1128/jvi.00373-20.
38. Parisien, J.P.; Lau, J.F.; Rodriguez, J.J.; Ulane, C.M.; Horvath, C.M. Selective STAT protein degradation induced by paramyxoviruses requires both STAT1 and STAT2 but is independent of alpha/beta interferon signal transduction. *J. Virol.* **2002**, *76*, 4190–4198.
39. Shaw, M.L.; García-Sastre, A.; Palese, P.; Basler, C.F. Nipah virus V and W proteins have a common STAT1-binding domain yet inhibit STAT1 activation from the cytoplasmic and nuclear compartments, respectively. *J. Virol.* **2004**, *78*, 5633–5641.
40. Erdős, G.; Dosztányi, Z. Analyzing Protein Disorder with IUPred2A. *Curr. Protoc. Bioinform.* **2020**, *70*, e99.
41. Lo, M.K.; Miller, D.; Aljofan, M.; Mungall, B.A.; Rollin, P.E.; Bellini, W.J.; Rota, P.A. Characterization of the antiviral and inflammatory responses against Nipah virus in endothelial cells and neurons. *Virology* **2010**, *404*, 78–88.
42. Martinez-Gil, L.; Vera-Velasco, N.M.; Mingarro, I. Exploring the Human-Nipah Virus protein–protein Interactome. *J. Virol.* **2017**, *91*, e01461.
43. Lange, A.; Mills, R.E.; Lange, C.J.; Stewart, M.; Devine, S.E.; Corbett, A.H. Classical nuclear localization signals: Definition, function, and interaction with importin alpha. *J. Biol. Chem.* **2007**, *282*, 5101–5105.
44. Stewart, M. Molecular mechanism of the nuclear protein import cycle. *Nat. Rev. Mol. Cell Biol.* **2007**, *8*, 195–208.
45. Goldfarb, D.S.; Corbett, A.H.; Mason, D.A.; Harreman, M.T.; Adam, S.A. Importin alpha: A multipurpose nuclear-transport receptor. *Trends Cell Biol.* **2004**, *14*, 505–514.
46. Garcia-Sastre, A. Mechanisms of inhibition of the host interferon alpha/beta-mediated antiviral responses by viruses. *Microbes Infect.* **2002**, *4*, 647–655.
47. Smith, K.M.; Tsimbalyuk, S.; Edwards, M.R.; Cross, E.M.; Batra, J.; Soares da Costa, T.P.; Aragao, D.; Basler, C.F.; Forwood, J.K. Structural basis for importin alpha 3 specificity of W proteins in Hendra and Nipah viruses. *Nat. Commun*. **2018**, *9*, 3703.
48. Kumar, K.P.; McBride, K.M.; Weaver, B.K.; Dingwall, C.; Reich, N.C. Regulated nuclear-cytoplasmic localization of interferon regulatory factor 3, a subunit of double-stranded RNA-activated factor 1. *Mol. Cell Biol.* **2000**, *20*, 4159–4168.
49. Zhu, M.; Fang, T.; Li, S.; Meng, K.; Guo, D. Bipartite Nuclear Localization Signal Controls Nuclear Import and DNA-Binding Activity of IFN Regulatory Factor 3. *J. Immunol.* **2015**, *195*, 289–297.
50. Seth, R.B.; Sun, L.; Chen, Z.J. Antiviral innate immunity pathways. *Cell Res.* **2006**, *16*, 141–147.
51. Braciale, T.J.; Hahn, Y.S. Immunity to viruses. *Immunol. Rev.* **2013**, *255,* 5–12.
52. Ivashkiv, L.B.; Donlin, L.T. Regulation of type I interferon responses. *Nat. Rev. Immunol.* **2014**, *14*, 36–49.
53. Gay, N.J.; Symmons, M.F.; Gangloff, M.; Bryant, C.E. Assembly and localization of Toll-like receptor signalling complexes. *Nat. Rev. Immunol.* **2014**, *14*, 546–558.

The authors and the Editorial Office would like to apologize for any inconvenience caused and state that the scientific conclusions are unaffected. The original publication has also been updated.
